# Laceration of the Cervic Uteri as a Cause of Post-Partum Hemorrhage

**Published:** 1880-09

**Authors:** 


### LACERATION OF THE CERVIX UTERI AS A CAUSE OF POST-PARTUM HEMORRHAGE.

A CASE of this kind is narrated by Dr. A. De la Roche. The patient was a primipara, aged twenty. The first stage of labor went on well, but in the second pains became feeble, and progress was arrested; therefore, sixteen hours after the beginning of labor, the forceps were applied, and delivery easily effected. Very little hemorrhage followed, and a quarter of an hour afterwards the placenta was delivered without trouble. Immediately this was done blood began to escape in abundance, but the uterus remained quite hard. The abdominal aorta was compressed and ergot given. At the end of twenty minutes compression was left off, and the hemorrhage recommenced, the patient falling into a state of syncope. The hand was then introduced, that the uterus might be compressed, and thus the existence of the cervical rent was discovered. The abdominal aorta was again compressed, and ice applied internally; but hemorrhage continued to recur at intervals, and it was two and a half hours before it was finally stopped. The slightness of the hemorrhage before the placenta was delivered, the author explains by supposing that the uterine contraction which expelled the placenta, also squeezed out clots which, until then, had kept the hemorrhage in abeyance.—*Lyon Medical.*

